# Theoretical Insights into Specific Ion Effects and Strong‐Weak Acid‐Base Rules for Ions in Solution: Deriving the Law of Matching Solvent Affinities from First Principles

**DOI:** 10.1002/cphc.202000644

**Published:** 2020-11-10

**Authors:** Ramón Alain Miranda‐Quintana, Jens Smiatek

**Affiliations:** ^1^ Department of Chemistry University of Florida Gainesville FL 32603 USA; ^2^ Institut für Computerphysik Universität Stuttgart 70569 Stuttgart Germany

**Keywords:** density functional calculations, Hofmeister series, ion pair formation, matching solvent affinities, specific ions effects

## Abstract

We present a detailed study of specific ion effects, volcano plots and the law of matching solvent affinities by means of a conceptual density functional theory (DFT) approach. Our results highlight that specific ion effects and the corresponding implications on the solvation energy are mainly due to differences in the electric chemical potentials and chemical hardnesses of the ions and the solvent. Our approach can be further used to identify reliable criteria for the validity of the law of matching solvent affinities. Basic expressions are derived, which allow us to study the limiting conditions for this empirical observation with regard to matching chemical reactivity indices. Moreover, we show that chaotropic and kosmotropic concepts and their implications for the stability of ion pairs are directly related to a generalized strong and weak acids and bases (SWAB) principle for ions in solution, which is also applicable to rationalize the shape of volcano plots for different solvents. In contrast to previous assumptions, all empirical findings can be explained by the properties of local solvent‐ion complexes which dominate the specific global behavior of ion pairs in solution.

## Graphical abstract

**Keep it local**: We present a detailed study of specific ion effects and the law of matching solvent affinities by means of conceptual density functional theory calculations. Our results underline the strong influence of the solvent in combination with the electronic chemical potential and the chemical hardness of the species. Further analytical considerations shed light on the principles of ion pair formation and ion pair dissociation with regard to a new formulation within the framework of strong and weak acids and bases.

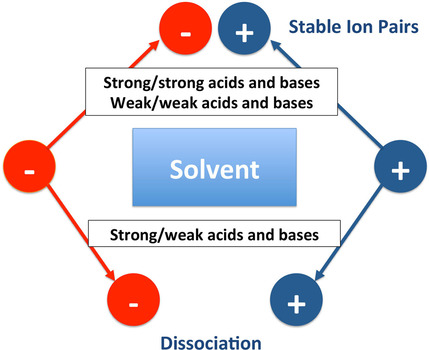

## 1 Introduction

Specific ion effects and the corresponding influence on solvent structure and dynamics attracted emerging attention in recent years.[^1^, ^2^, ^3^, ^4^, ^5^, ^6^, ^7^, ^8^, ^9^, ^10^, ^11^, ^12^] The interest in a deeper understanding of these effects is mainly due to the broad impact on various research disciplines and fields of application. In more detail, specific ion effects manifest themselves in different solvation energies for certain ion pairs as well as an individual binding and aggregation behavior in presence of different solvents and solutes.[^1^, ^2^, ^3^, ^7^, ^13^, ^14^, ^15^, ^16^, ^17^, ^18^] In consequence, the aforementioned effects influence and modify various molecular mechanisms such as differences in ion pairing behavior, counterion condensation around polyelectrolytes, stability of protein formulations and the conductivity of electrolyte solutions.[^2^, ^7^, ^16^, ^17^, ^18^, ^19^, ^20^, ^21^, ^22^]

Despite the growing interest, the recent level of theoretical understanding regarding the underlying mechanisms is rather low. Advanced explanations focused on a combination of electrostatic interactions between the ions and short‐range dispersion, polarization and solvation contributions.[^4^, ^6^, ^7^, ^10^, ^19^, ^20^, ^23^, ^24^, ^25^, ^26^] In addition, previous work also has highlighted the advantages of considering thermodynamic arguments in combination with finite ion sizes and discrete hydration effects,[^27^, ^28^] but the molecular mechanisms that lead to certain observations like reversed solvent‐ and surface‐initiated specific ion effects or pH value‐related mechanisms are not yet fully clarified.[^6^, ^10^, ^29^, ^30^] In combination with these novel insights, it was also recently shown that specific ion effects are abundant in all solvents and are not restricted to aqueous solutions as previously assumed.[^8^, ^9^, ^12^, ^31^]

A reasonable experimental approach to study specific ion effects in various solvents are measurements of solvation energies and their interpretation in terms of volcano plots.[^2^, ^7^, ^12^, ^31^] The corresponding analysis for aqueous solutions under consideration of stable and weak ion pairs lead to the definition of kosmotropic and chaotropic ions.[^3^, ^7^, ^32^] Here, it is assumed that kosmotropic ions as water structure makers stabilize the surrounding solvent structure while chaotropic ions as water structure breakers lead to a perturbation of the local solvent arrangement.[^3^, ^32^] Usually, kosmotropes are represented by small ions with high surface charge density like F^−^ or Li^+^ in contrast to larger and thus chaotropic ions with lower surface charge densities like SCN^−^ or I^−^. In terms of stable and instable pairs, it has been observed that ion pairs with high and low surface charge density of the individual species form the most stable combinations. These observations lead to the formulation of the „law of matching water affinities“[^2^, ^7^, ^31^, ^33^] which relies on the empirical finding, that the stability of ion pairs can be attributed to the influence of the individual ions on the water structure. In more detail, the corresponding law states that oppositely charged ions with similar water affinities, meaning comparable hydration enthalpies, tend to associate and to form stable contact ion pairs. In addition to aqueous solutions, previous experimental findings also demonstrated the presence of specific ions effects and the validity of the law of matching water affinities for a series of protic and aprotic solvents,[^9^, ^12^] such that it was generalized to a „law of matching solvent affinities“. Notably, the law of matching water or solvent affinities was also used to provide a rationale for the the salting‐in and salting‐out behavior of proteins in presence of various ions.[^2^, ^7^, ^34^] Although not rigorously proven, the application of this empirical concept is widely accepted in various fields of research.[^2^, ^3^, ^4^, ^7^]

Besides the modification of electrostatic theories by consideration of polarization, dispersion as well as solvation contributions, recent papers highlighted the benefits of a conceptual density functional theory (DFT) approach in order to study specific ion effects.[^11^, ^12^, ^21^, ^35^, ^36^] Conceptual DFT is an analytic approach which was often used to provide rationales for chemical principles, reaction mechanisms, chemical reactivities as well as molecular properties.[^36^, ^37^, ^38^, ^39^, ^40^, ^41^, ^42^, ^43^, ^44^, ^45^, ^46^] The validity of a modified conceptual DFT approach for the study of various ion properties in distinct solutions was recently demonstrated.[^11^, ^12^] The results revealed a reasonable agreement with experimental findings and the corresponding slight deviations can be attributed to the known approximations of the approach.^[12]^

In this article, we present a first principles conceptual DFT approach in order to provide explanations for the occurrence of specific ion effects and the law of matching solvent affinities. In previous studies, we demonstrated the benefits of our approach for the numerical calculation of ion‐pair solvation energies as well as donor numbers in good agreement with experimental findings.[^11^, ^12^] Here, we use an extended version of the framework to derive and to provide analytic expressions in order to elucidate the fundamental principles of specific ion effects as well as the behavior of ion pairs in solution. The results of this article shed more light on the nature of interactions as well as the interplay between ions and solvents to rationalize certain experimental observations. In detail, our theoretical analysis in terms of chemical reactivity indices such as chemical hardnesses and electronegativities provides molecular explanations for the special shape of volcano plots as well as the specific properties of ion pairs in different solvents. With regard to this point, we define limiting conditions for the validity of the law of matching solvent affinities which reveal that this empirical concept has to be replaced by a generalized strong and weak acids and bases (SWAB) principle. Furthermore, we also demonstrate the applicability of our approach for the study of kosmotropic and chaotropic effects. The corresponding relations rationalize recent experimental findings as well as general principles for ions in solution.

The paper is organized as follows. In the next section, we present a basic overview on the fundamental principles of conceptual DFT for the study of solvation mechanisms. The mathematical framework of our approach and its application to study the governing principles behind specific ion effecs, the law of matching solvent affinities and volcano plots are in detail discussed in section 3. A summary and a conclusion of our results is provided in the last section.

## 2 Theoretical Background

### 2.1 Conceptual Density Functional Theory

Over the last four decades, conceptual DFT calculations were used successfully in various contexts.[^37^, ^38^, ^39^, ^40^, ^41^, ^42^, ^43^] Due to its original intent,[^37^, ^38^] conceptual DFT establishes a basic understanding of reactive behavior and can also be used to rationalize well‐known rules like the hard/soft acids and bases (HSAB), maximum hardness, minimum electrophilicity, and the „$\left|\Delta \mu \right|$ big is good“ principles.[^36^, ^47^, ^48^, ^49^, ^50^, ^51^, ^52^, ^53^, ^54^, ^55^, ^56^] Previous efforts in this direction[^38^, ^45^, ^49^, ^57^, ^58^] already revealed the many benefits of this straightforward approach.[^11^, ^21^, ^44^, ^50^, ^58^, ^59^] Here, we outline the basic ideas and main concepts.

### 2.2 Chemical Reactivity Indices

As most fundamental expression,[^37^, ^38^, ^45^, ^49^, ^57^, ^58^] the electronic chemical potential μ of an atom or molecule can be defined as the derivative of the total electronic energy *E* under a constant nuclear or external potential $\nu \left(r\right)$ in accordance with

(1) $$\mu ={\left(\frac{\partial E}{\partial N}\right)}_{\nu \left(r\right)},$$

where *N* denotes the actual number of electrons of the molecule.[^37^, ^38^, ^39^, ^40^, ^42^] In addition, the electronegativity *χ* is defined by[^37^, ^38^, ^39^, ^42^]

(2) $$\chi =-\mu =-{\left(\frac{\partial E}{\partial N}\right)}_{\nu \left(r\right)},$$

which represents a more robust mathematical expression when compared to previous empirical assumptions.^[60]^ With regard to these expressions, the energy change of isolated molecules upon electronic perturbation can be written as a Taylor series according to

(3) $$\Delta E={\left(\frac{\partial E}{\partial N}\right)}_{\nu \left(r\right)}\Delta N+\frac{1}{2}{\left(\frac{\partial^{2}E}{\partial N^{2}}\right)}_{\nu \left(r\right)}(\Delta N{)}^{2}=-\chi \Delta N+\frac{1}{2}\eta (\Delta N{)}^{2}$$

where the chemical hardness *η* is introduced as[^38^, ^39^, ^40^, ^41^]

(4) $$\eta ={\left(\frac{\partial^{2}E}{\partial N^{2}}\right)}_{\nu \left(r\right)}={\left(\frac{\partial \mu }{\partial N}\right)}_{\nu \left(r\right)}=-{\left(\frac{\partial \chi }{\partial N}\right)}_{\nu \left(r\right)}$$

which can be regarded as a resistance of the chemical species against electronic changes.^[39]^ In terms of the Koopmans theorem for Hartree–Fock orbitals, or Janak's theorem for Kohn‐Sham orbitals,^[61]^ the values for the chemical hardness and the electronegativity can be approximated by[^40^, ^41^, ^42^, ^58^, ^60^, ^62^]

(5) $$\chi =\frac{1}{2}\left(I+A\right)=-\frac{1}{2}\left(E_{\mathrm{HOMO}}+E_{\mathrm{LUMO}}\right)$$

and

(6) $$\eta ≃I-A=E_{\mathrm{LUMO}}-E_{\mathrm{HOMO}},$$

where $I=-E_{\mathrm{HOMO}}$ and $A=-E_{\mathrm{LUMO}}$ denote the vertical ionization potential and the vertical electron affinity, respectively, and *E*_HOMO_ and *E*_LUMO_ the corresponding values for the energies associated with the highest occupied molecular orbital (*E*_HOMO_) and the lowest unoccupied molecular orbital (*E*_LUMO_), respectively. In Eqn. (5) and Eqn. (6), it is assumed that the energy levels do not change upon electron loss or uptake. A more refined approach is presented in Ref. [63], where it is discussed that for a molecule in its ground‐state, the number of electrons is *N*_0_, such that electron uptake and loss are denoted by *N*_0_+1 and *N*_0_−1, respectively. The electronic chemical potential for electron uptake thus reads^[63]^

(7) $$\mu^{+}={\left(\frac{\partial E}{\partial N}\right)}_{\nu \left(r\right)}^{+}=E_{N_{0}+1}-E_{N_{0}}=-A$$

whereas the electron loss can be written as

(8) $$\mu^{-}={\left(\frac{\partial E}{\partial N}\right)}_{\nu \left(r\right)}^{-}=E_{N_{0}}-E_{N_{0}-1}=-I.$$

Here, it is assumed that $(\partial E/\partial N{)}_{\nu \left(r\right)}\approx \Delta E/\Delta N$ . Hence, the Mulliken definition^[60]^ in Eqn. (5) can also be derived by a more sophisticated finite‐difference approximation as outlined in Refs. [11, 38, 40, 50, 63]. An analogous procedure can also be applied for the detailed derivation of the chemical hardness.[^11^, ^40^, ^63^]

### 2.3 Reaction Energies

One of the further benefits of conceptual DFT besides the introduction of chemical reactivity indices is its rigorous definition of energy changes upon chemical reactions.[^49^, ^50^, ^58^] Hence, any chemical reaction between two species A and B in terms of

(9) A+B→AB

can be studied straightforwardly by introducing the chemical reactivity indices in terms of electronegativities and chemical hardnesses. With regard to this point, it was shown,[^11^, ^39^, ^45^] that the corresponding half‐reaction energy associated with the reaction in Eqn. (9) reads

(10) $$\Delta E_{\mathrm{AB}}=-\frac{1}{2}\frac{(\chi_{A}-\chi_{B}{)}^{2}}{\eta_{A}+\eta_{B}}$$

with the electronegativities $\chi_{A},\chi_{B}$ and hardnesses $\eta_{A},\eta_{B}$ of species A and B. For the calculation of equilibrium energies with regard to forward and backward reactions, Eqn. (9) can be extended by consideration of additional half‐reactions.[^49^, ^50^] A comparable concept was also used for the calculation of solvation energies and their differences for ion pairs in various solvents. The corresponding results revealed a good agreement with experimental data.[^11^, ^12^]

### 2.4 Solvation of Ion Pairs

In the following, we derive basic expressions of the conceptual DFT approach for the computation of ion solvation energies as previously introduced in Refs. [11, 12]. As a prerequisite, it is assumed that the individual chemical hardnesses $\eta_{C}$ , $\eta_{A}$ as well as the corresponding electronegativities $\chi_{C}$ , $\chi_{A}$ of the cation (index'C’) and anion (index'A’) are known. With regard to the law of matching solvent affinities, it was shown that certain solvents (index'S’) with electronegativity $\chi_{S}$ and chemical hardness $\eta_{S}$ are able to maximize the full solvation enthalpy for a given ion pair.^[12]^ Here, it is assumed that the solvation enthalpies can be approximated by solvation energies as computed from conceptual DFT calculations.^[12]^ Hence, the full solvation energy of an ion pair in a solvent reads

(11) $$\Delta \Delta E_{\mathrm{solv}}=\Delta E_{\mathrm{CS}}+\Delta E_{\mathrm{AS}}-\Delta E_{\mathrm{CA}}$$

as a sum of the individual reaction energies between the cation and the solvent

(12) $$\Delta E_{\mathrm{CS}}=-\frac{1}{2}\frac{{\left(\chi_{C}-\chi_{S}\right)}^{2}}{\eta_{C}+\eta_{S}},$$

between the anion and the solvent

(13) $$\Delta E_{\mathrm{AS}}=-\frac{1}{2}\frac{{\left(\chi_{A}-\chi_{S}\right)}^{2}}{\eta_{A}+\eta_{S}},$$

and between the cation and the anion

(14) $$\Delta E_{\mathrm{CA}}=-\frac{1}{2}\frac{{\left(\chi_{C}-\chi_{A}\right)}^{2}}{\eta_{C}+\eta_{A}}$$

in close analogy to the interpretation of chemical half reactions according to Eqn. (9). In particular, Eqn. (11) does not relate to specific ion pair states, such that it can be understood as an average over contact ion pairs, solvent shared ion pairs, solvent separated ion pairs and ion aggregates.[^15^, ^17^] This can be justified by the rapid transitions between the states and the corresponding metastable behavior.^[15]^ Dynamic transitions between the individual ion states usually occur on the time scale of a few nanoseconds or even shorter, such that the resulting experimental solvation enthalpy of an infinitely diluted ion pair can be regarded as a weighted sum of the individual energetic contributions. Our approach focuses explicitly on this infinite dilution limit and hence ignores all higher ionic correlation effects. Notably, ions in solution interact via unscreened long‐range electrostatic interactions. With regard to previous discussions,[^11^, ^49^, ^50^] it was shown that all electrostatic, polarization and dispersion interactions can be interpreted as higher order effects which are usually smaller than $\Delta \Delta E_{\mathrm{solv}}$ (Eqn. (11)), and thus can be safely ignored. Only for very small and hard ions, notable contributions from electrostatic interactions have to be taken into consideration as discussed in more detail in Refs. [49, 50].

The difference in the cation and anion solvation energies can be calculated via

(15) $$\Delta \Delta E_{\mathrm{AC}}=\Delta E_{\mathrm{AS}}-\Delta E_{\mathrm{CS}}$$

under consideration of Eqns. (13) and (14). Notably, it was shown that $\Delta \Delta H_{\mathrm{solv}}∼\Delta \Delta E_{\mathrm{solv}}$ and $\Delta \Delta H_{\mathrm{AC}}∼\Delta \Delta E_{\mathrm{AC}}$ , which means that the corresponding Eqns. (11) and (15) are reasonable approximations for the solvation enthalpies and the corresponding solvation enthalpy differences of the ions.^[12]^ With reference to their general meaning, both enthalpy values are main parameters for the study of specific ion effects in terms of volcano plots.

**Figure 1:**
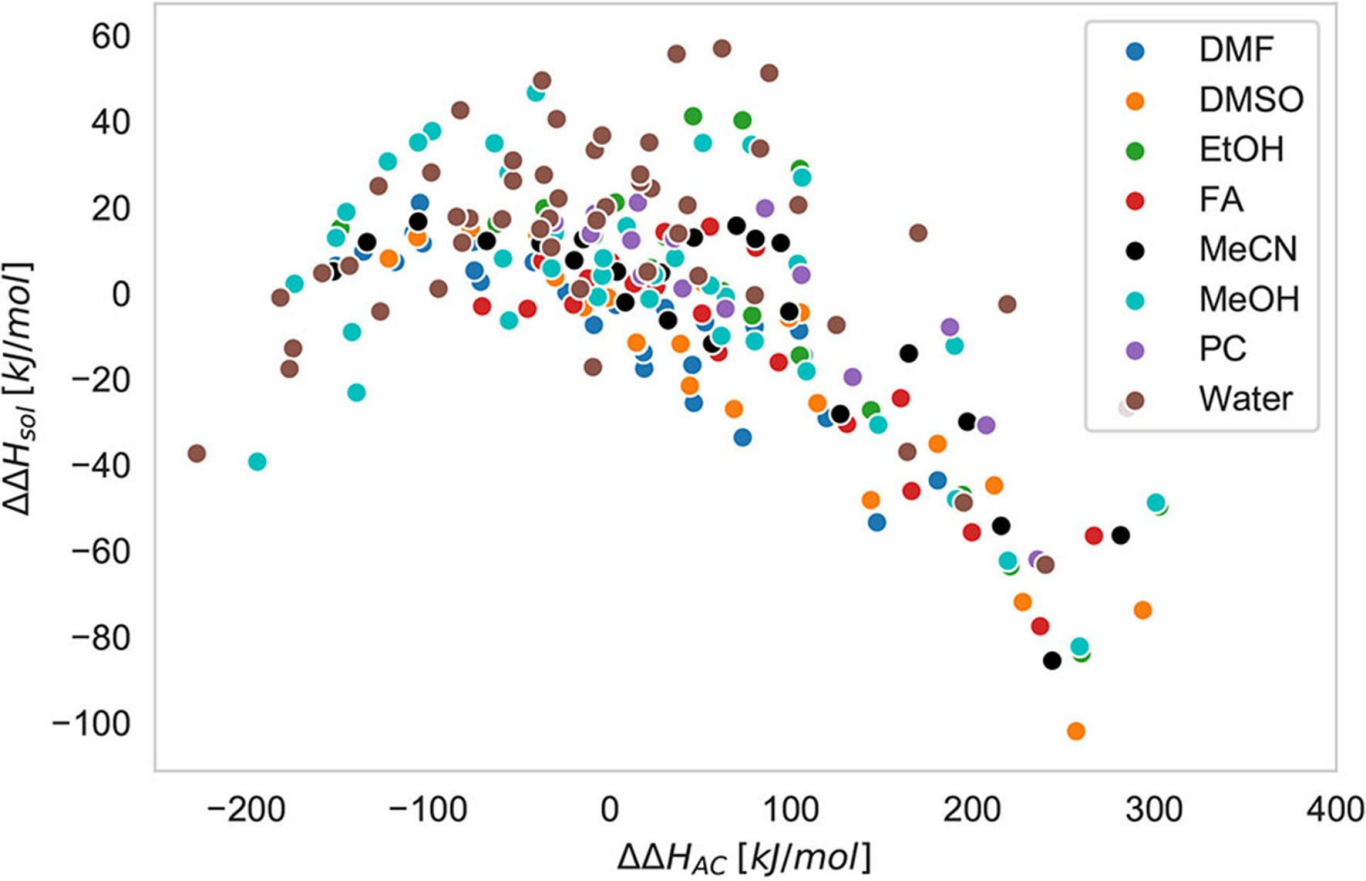
Volcano plot for various ion pairs including all alkali and halide ions in the solvents dimethyl formamide (DMF), dimethyl sulfoxide (DMSO), ethanol (EtOH), formamide (FA), acetamide (ACE), methanol (MeOH), propylene carbonate (PC) and water. The experimental values for the differences in the ion solvation enthalpies $\Delta \Delta H_{\mathrm{AC}}$ and the full solvation enthalpies $\Delta \Delta H_{\mathrm{sol}}$ were taken from Refs. [3, 31].

In more detail, volcano plots are commonly used to verify the law of matching solvent affinities as well as the presence of specific ion effects. If one combines the full solvation enthalpy $\Delta \Delta H_{\mathrm{sol}}$ of an ion pair in infinite dilution in a specific solvent with the difference in the individual solvation enthalpies for cations and anions according to

(16) $$\Delta \Delta H_{\mathrm{AC}}=\Delta H_{\mathrm{AS}}-\Delta H_{\mathrm{CS}}$$

where $\Delta H_{\mathrm{AS}}$ denotes the solvation enthalpy of the anion and $\Delta H_{\mathrm{CS}}$ the solvation enthalpy of the cation, respectively, one can observe a volcano plot‐like behavior for most solvent‐ion pair combinations where the largest positive values for $\Delta \Delta H_{\mathrm{solv}}$ can be observed around $\Delta \Delta H_{\mathrm{AC}}\approx 0$ (Figure 1). The corresponding experimental values are usually obtained by calorimetric experiments that measure the heat of solvation (solvation enthalpy) at distinct finite ion pair concentrations, which are then extrapolated to obtain the corresponding value at the infinite dilution limit.^[3]^ In terms of $\Delta \Delta H_{\mathrm{AC}}$ , gaseous ions are gradually brought into contact with the corresponding solvent molecules, which provides the corresponding solvation enthalpy difference of the individual ions.^[3]^

With regard to the shape of the volcano plot in Figure 1, it has to be noted that the definition of a „volcano plot“ has to be slightly modified for non‐aqueous electrolyte solutions when compared to aqueous systems.[^31^, ^33^] In agreement with Ref. [31], we define a volcano plot as a scatter plot of the full solvation enthalpies of ion pairs and the individual solvation enthalpies of the cations and the anions, respectively, which shows a maximum in a quite narrow interval around vanishing anion and cation solvation enthalpy differences. It has to be noted that the expected linear and symmetrical decrease around the maximum values for $\Delta \Delta H_{\mathrm{solv}}$ usually change for non‐aqueous solvents when compared to the original definition. Hence, we use the term volcano plot in a historical context, although the meaning often becomes not immediately apparent due to the lack of complete symmetry.^[31]^ Despite these shortcomings for non‐aqueous solvents, a steep decrease of the full solvation enthalpy can be observed for $\left|\Delta \Delta H_{\mathrm{AC}}\right|>0$ . Although one usually observes a stronger decrease in a certain direction,^[31]^ *e. g*., a larger change of $\Delta \Delta H_{\mathrm{solv}}$ either for $\Delta \Delta H_{\mathrm{AC}}<0$ or $\Delta \Delta H_{\mathrm{AC}}>0$ , the most stable ion pairs reveal vanishing values of $\Delta \Delta H_{\mathrm{AC}}$ as required by the law of matching solvent affinities.^[31]^

## 3 Conceptual DFT: Specific Ion Effects and the Law of Matching Solvent Affinities

This section presents the main equations of our proposed conceptual DFT approach in order to rationalize specific ion effects in various solvents, the properties of volcano plots as well as necessary conditions for the validity of the law of matching solvent affinities. Thus, we rely strongly on the validity of the conceptual DFT approach for ions in solution,[^11^, ^12^] such that it is assumed that the computed solvation energies are reasonable approximates for the experimental solvation enthalpies.

### 3.1 Conditions for the Solvent: Maximum Solvation Energies

For any given ion pair with known electronegativities and chemical hardnesses, one can compute the electronegativity $\chi_{S}^{\max}$ and the chemical hardness $\eta_{S}^{\max}$ of the solvent which maximizes the full solvation energy (Eqn. 11)) according to

(17) $$\frac{\partial }{\partial \chi_{S}}\Delta \Delta E_{\mathrm{solv}}=0$$

and

(18) $$\frac{\partial }{\partial \eta_{S}}\Delta \Delta E_{\mathrm{solv}}=0.$$

Notably, the derivative in Eqn. (18) according to Eqn. (11) yields

(19) $$\frac{\partial }{\partial \eta_{S}}\Delta \Delta E_{\mathrm{solv}}=\frac{1}{2}\frac{{\left(\chi_{A}-\chi_{S}\right)}^{2}}{{\left(\eta_{C}+\eta_{S}\right)}^{2}}+\frac{1}{2}\frac{{\left(\chi_{C}-\chi_{S}\right)}^{2}}{{\left(\eta_{A}+\eta_{S}\right)}^{2}}=0$$

which only vanishes if $\chi_{S}=\chi_{C}=\chi_{A}$ . With regard to recent considerations,[^11^, ^12^, ^50^] one usually assumes the orders

(20) $$\chi_{C}>\chi_{S}>\chi_{A}>0$$

and

(21) $$\eta_{C}>\eta_{S}>\eta_{A}>0$$

which reveals that $\partial \Delta \Delta E_{\mathrm{solv}}/\partial \eta_{S}>0$ holds in any case. Therefore, it can be concluded that the full solvation energy is monotonously increasing with increasing hardness to the maximum value

(22) $$\lim_{\eta_{S}^{\max}\to \infty }\Delta \Delta E_{\mathrm{solv}}=\frac{1}{2}\frac{{\left(\chi_{C}-\chi_{A}\right)}^{2}}{\eta_{C}+\eta_{A}}$$

after consideration of Eqns. (11), (12), (13) and (14). These findings are in good agreement with recent articles, where it was discussed that a solvated ion can be regarded as a charge transfer complex[^11^, ^12^, ^64^, ^65^] due to strong electronic polarization effects with the surrounding solvent molecules. With regard to Eqn. (22), it becomes clear that the presence of a polarizable solvent around the ions is of fundamental importance in order to lower the full solvation energy.

In addition to Eqn. (18), the derivative of Eqn. (17) reads

(23) $$\frac{\partial }{\partial \chi_{S}}\Delta \Delta E_{\mathrm{solv}}=\frac{\left(\chi_{A}-\chi_{S}^{\max}\right)}{\left(\eta_{C}+\eta_{S}\right)}+\frac{\left(\chi_{C}-\chi_{S}^{\max}\right)}{\left(\eta_{A}+\eta_{S}\right)}=0$$

which further gives

(24) $$\chi_{S}^{\max}=\frac{\chi_{C}+\gamma \chi_{A}}{1+\gamma }$$

with

(25) $$\gamma =\frac{\eta_{C}+\eta_{S}}{\eta_{A}+\eta_{S}}>0$$

after suitable rearrangement. Notably, Eqn. (24) is closely related to previously published expressions for perturbed electronegativities in terms of modified HOMO and LUMO energies upon reaction and also satisfies the presented orders in Eqn. (20) and Eqn. (21).[^11^, ^44^, ^45^] The insertion of Eqn. (24) into Eqn. (11) leads to the maximum full solvation energy

(26) $$\Delta \Delta E_{\mathrm{solv}}^{\max}\left(\chi_{S}^{\max}\right)=\Delta E_{\mathrm{CS}}\left(\chi_{S}^{\max}\right)+\Delta E_{\mathrm{AS}}\left(\chi_{S}^{\max}\right)-\Delta E_{\mathrm{CA}}\left(\chi_{S}^{\max}\right)$$

for any given ion pair. Notably, the hardness values of most common solvents are $\eta_{S}=3-8$ eV^[12]^ which implies that the full solvation energy is mainly dominated by the electronegativity of the solvent ($𝒪\left(1/\eta_{S}\right)\ll 𝒪\left(\chi_{S}^{2}\right)$ ). Here, we do not introduce any requirements for the chemical hardness of the solvent, but if we assume an infinite chemical hardness as limiting condition, it follows from Eqn. (24) that

(27) $$\lim_{\eta_{S}^{\max}\to \infty }\chi_{S}^{\max}=\frac{1}{2}\left(\chi_{C}+\chi_{A}\right)$$

which is comparable to the expression derived in Ref. [12]. With regard to this interpretation and previous considerations,^[11]^ it is beneficial to employ acidic or basic solvents which differ significantly in their binding energy to the individual ions in terms of $\chi_{S}\gg \left(1/2\right)\left(\chi_{C}+\chi_{A}\right)$ or $\chi_{S}\ll \left(1/2\right)\left(\chi_{C}+\chi_{A}\right)$ , respectively, such that one ionic species is favored upon solvation. These findings have important implications for electrolyte solutions in battery research with fixed cations like Li^+^ or Na^+^ and a limited set of suitable solvents with high electrochemical stability windows.^[17]^

### 3.2 Conditions for the Ions: Maximum Solvation Energies

In addition to the identification of suitable solvents for given ion pairs, it is also possible to identify ion pairs for given solvents whose combination results in maximum full solvation energies. For purposes of more straightforward calculations, we re‐write the ion electronegativities by

(28) $$\chi_{C}=\chi_{S}+\delta_{C}>0$$

(29) $$\chi_{A}=\chi_{S}-\delta_{A}>0$$

as well as

(30) $$\chi_{C}-\chi_{A}=\delta_{C}+\delta_{A}>0$$

which satisfy Eqn. (20). With regard to maximum conditions for the cation, it follows

(31) $$\frac{\partial }{\partial \chi_{C}}\Delta \Delta E_{\mathrm{solv}}=0$$

as well as

(32) $$\frac{\partial }{\partial \eta_{C}}\Delta \Delta E_{\mathrm{solv}}=0$$

in agreement with our previous considerations for the solvent. Let us start with the optimization of the hardness of the cation

(33) $$\frac{\partial }{\partial \eta_{C}}\Delta \Delta E_{\mathrm{solv}}=0\Leftrightarrow \frac{\delta_{C}}{\eta_{C}+\eta_{S}}-\frac{\delta_{C}+\delta_{A}}{\eta_{A}+\eta_{C}}=0$$

which leads to

(34) $$\eta_{C}^{\max}=\frac{\eta_{A}\delta_{C}-\eta_{S}\left(\delta_{C}+\delta_{A}\right)}{\delta_{A}}.$$

However, according to Eqns. (28), (29) and (20), we defined $\delta_{A}>0$ and $\delta_{C}>0$ , such that we can easily see according to Eqn. (21) that

(35) $$\eta_{A}\delta_{C}-\eta_{S}\left(\delta_{C}+\delta_{A}\right)=\delta_{C}\left(\eta_{A}-\eta_{S}\right)-\delta_{A}\eta_{S}<0$$

which implies that

(36) $$\eta_{C}^{\max}<0,$$

thereby contradicting Eqn. (21) and the fact that the hardness must have a positive value because of the convexity of the electron energy.^[66]^ This situation is similar to that encountered in the case of the optimization of the hardness of the solvent. Despite the invalidity of Eqn. (36), it follows for the general trend of Eqn. (33) that

(37) $$\frac{\partial }{\partial \eta_{C}^{\max}}\Delta \Delta E_{\mathrm{solv}}<0,$$

which reveals that the maximum solvation energy will be achieved when the hardness of the cation is as small as possible.

We can follow a similar reasoning for the optimization of the hardness of the anion, noting that

(38) $$\frac{\partial }{\partial \eta_{A}}\Delta \Delta E_{\mathrm{solv}}=0\Leftrightarrow \frac{\delta_{A}}{\eta_{A}+\eta_{S}}-\frac{\delta_{C}+\delta_{A}}{\eta_{A}+\eta_{C}}=0$$

from where it follows that

(39) $$\eta_{A}^{\max}=\frac{\eta_{C}\delta_{A}-\eta_{S}\left(\delta_{C}+\delta_{A}\right)}{\delta_{C}}>0.$$

Here, $\delta_{C}>0$ by construction (Eqn. (21)), however, unlike for the cation, one can see that the nominator of Eqn. (39) has a positive value in terms of

(40) $$\left(\eta_{C}-\eta_{S}\right)\delta_{A}-\eta_{S}\delta_{C}>0.$$

such that we can optimize the hardness of the anion through a value determined by Eqn. (39).

In order to study the properties of varying cation electronegativities, we re‐write the full solvation energy (Eqn. (11)) as a function of Eqns. (28), (29) and (30) according to

(41) $$\Delta \Delta E_{\mathrm{solv}}=-\frac{1}{2}\frac{\delta_{C}^{2}}{\eta_{C}+\eta_{S}}-\frac{1}{2}\frac{\delta_{A}^{2}}{\eta_{A}+\eta_{S}}+\frac{1}{2}\frac{{\left(\delta_{C}+\delta_{A}\right)}^{2}}{\eta_{C}+\eta_{A}}$$

which yields after suitable transformation of Eqn. (31)

(42) $$\frac{\partial \Delta \Delta E_{\mathrm{solv}}}{\partial \delta_{C}}\frac{\partial \delta_{C}}{\partial \chi_{C}}=-\frac{\delta_{C}}{\eta_{C}+\eta_{S}}+\frac{\left(\delta_{C}+\delta_{A}\right)}{\eta_{C}+\eta_{A}}=0.$$

A comparable expression can be found for the anions and $\delta_{A}$ in terms of

(43) $$\frac{\partial \Delta \Delta E_{\mathrm{solv}}}{\partial \delta_{A}}\frac{\partial \delta_{A}}{\partial \chi_{A}}=-\frac{\delta_{A}}{\eta_{A}+\eta_{S}}+\frac{\left(\delta_{C}+\delta_{A}\right)}{\eta_{C}+\eta_{A}}=0$$

and if one combines Eqn. (42) and Eqn. (43), it follows

(44) $$\frac{\delta_{C}}{\eta_{C}+\eta_{S}}=\frac{\delta_{A}}{\eta_{A}+\eta_{S}}.$$

After usage of Eqn. (25), one obtains

(45) $$\delta_{C}=\gamma \delta_{A}$$

under the condition that the cation and anion electronegativities are optimized simultaneously. Insertion of the expressions above into Eqn. (42) or Eqn. (43), respectively, yields

(46) $$\delta_{C}\left(-\frac{1}{\eta_{C}+\eta_{S}}+\frac{1+\gamma }{\gamma \left(\eta_{A}+\eta_{C}\right)}\right)=0$$

and

(47) $$\delta_{A}\left(-\frac{1}{\eta_{A}+\eta_{S}}+\frac{1+\gamma }{\eta_{A}+\eta_{C}}\right)=0$$

which both need to be satisfied. In consequence and with regard to the ordering condition for the chemical hardnesses in Eqn. (21), both equations are only valid for $\delta_{C}=\delta_{A}=0$ which means that $\chi_{C}=\chi_{S}=\chi_{A}$ in contrast to Eqn. (20). Thus, a simultaneous optimization of the full solvation energy by variation of $\chi_{A}$ and $\chi_{C}$ is not possible. In addition, a separate optimization for the cation in terms of Eqn. (42) gives

(48) $$\delta_{C}=\delta_{A}\left(\frac{\eta_{C}+\eta_{S}}{\eta_{A}-\eta_{S}}\right)<0$$

with a negative value due to Eqn. (21) and Eqn. (29). In consequence, an increase of the cation electronegativity does not lead to a higher full solvation energy. In contrast, an increase of the anion electronegativity in terms of Eqn. (43) yields

(49) $$\delta_{A}^{\max}=\delta_{C}\left(\frac{\eta_{A}+\eta_{S}}{\eta_{C}-\eta_{S}}\right)>0$$

which is a positive value due to Eqn. (21) and Eqn. (28). As we have shown, we cannot directly maximize the solvation energy by optimizing the hardness or the electronegativity of the cation. On the other hand, we can actually maximize the solvation energy by optimizing the hardness or the electronegativity of the anion, as shown in Eqns. (39) and (49).

As a last point, one may ask if a simultaneous change of the electronegativity and the chemical hardness for a certain ion leads to larger full solvation energies? In fact, further evaluation of Eqn. (32) provides>

(50) $$\frac{\partial }{\partial \eta_{C}}\Delta \Delta E_{\mathrm{solv}}=\frac{\delta_{C}^{2}}{{\left(\eta_{C}^{\max}+\eta_{S}\right)}^{2}}-\frac{{\left(\delta_{C}+\delta_{A}\right)}^{2}}{{\left(\eta_{A}+\eta_{C}^{\max}\right)}^{2}}=0$$

which is equivalent to Eqn. (42) and therefore establishes a single equation and condition for the electronegativity and chemical hardness. Thus, both properties cannot be changed simultaneously in order to reach higher full solvation energies. Analogous expressions also hold for the anions.

### 3.3 Summary of Previous Findings

Our previous findings can be summarized as follows. For given ion pairs, one can identify an expression for the solvent electronegativity (Eqn. (24)) which maximizes the full solvation energy. Moreover, by increasing the solvent hardness, one can obtain higher full solvation energies with a maximum value as defined in Eqn. (27). A simultaneous change of the electronegativity and chemical hardness of the solvent for higher full solvation energies is not possible.

**Figure 2:**
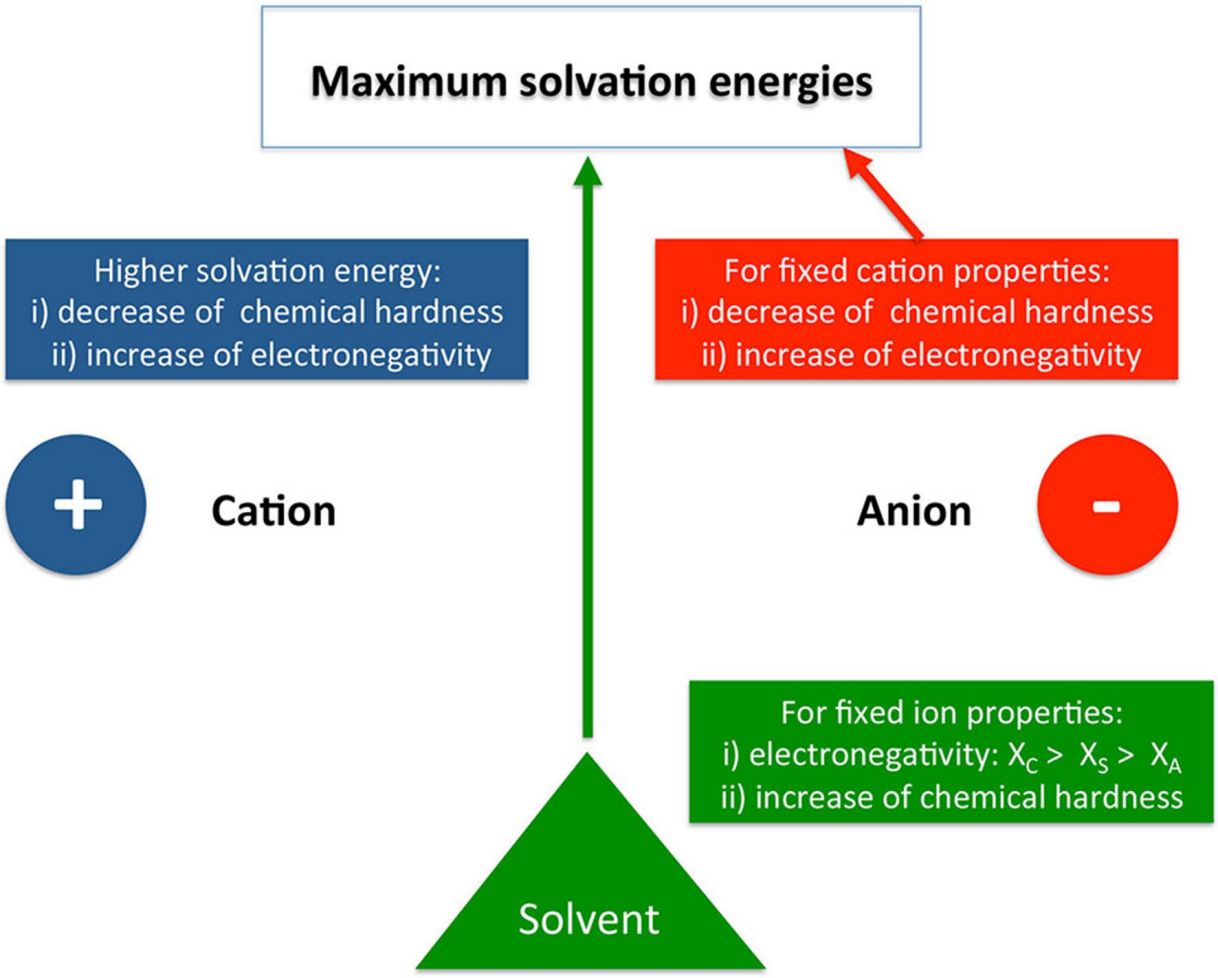
Visual representation of solvent and ion conditions which maximize the solvation energy in terms of most stable ion pairs. Notably, only a variation of the anion for given solvent and cation values as well as the variation of the solvent for given ion pairs results in maximum solvation energies. The variation of the cation properties only increases the full solvation energy, but not to a maximum value.

For given solvents, one can also identify the corresponding chemical reactivity indices of the ions which increase the full solvation energy. Trying to directly optimize the hardness and/or the electronegativity of the cation can lead to contradictions, and one can only conclude that one should expect an increase in the solvation energy by either increasing the electronegativity or decreasing the hardness of the cation. Somehow similarly, a simultaneous optimization of the electronegativity and hardness of the anion leads to inconsistencies. However, as shown in Eqns. (39) and (49), the full solvation energy can be maximized by changing the anions chemical hardness and electronegativity separately. Furthermore, a a combination of Eqn. (49) with Eqns. (28), (29) and (30) reads

(51) $$\chi_{A}^{\max}=\chi_{S}-\left(\chi_{C}-\chi_{S}\right)\left(\frac{\eta_{A}+\eta_{S}}{\eta_{C}-\eta_{S}}\right)$$

as a necessary relation for a maximum full solvation energy. A visual representation of these findings is provided in Figure 2.

Notably and for most applications, research focuses on less stable ion pairs which reveal high solubilities.^[17]^ In terms of such requirements, one can state that solvents should have low chemical hardnesses and their electronegativities should differ significantly from Eqn. (24). Moreover, a change of the cation leads to a varying behavior, such that lowering the anion chemical hardness as well as looking for anions whose electronegativities differ from Eqn. (51) is more promising. The corresponding findings reveal that specific ion effects are mainly due to the chemical reactivity indices of the ions and the solvent. In contrast to other approaches, the respective expressions provide useful criteria to identify suitable solvent‐ion pair combination in order to meet individual purposes.

### 3.4 A Generalized SWAB Principle for Ions in Solution

The previous expressions can also be used to study the validity of the law of matching solvent affinities, which states that maximum full solvation enthalpies are reserved for ion pairs with vanishing differences in the ion solvation enthalpies.[^2^, ^3^, ^7^, ^9^, ^12^] In addition to our previous focus on maximum full solvation energies, one also has to consider vanishing values for $\Delta \Delta E_{\mathrm{AC}}$ as a second boundary condition. Strictly speaking, the law of matching solvent affinities is only valid if maximum solvation energies for ion pairs are located at $\Delta \Delta E_{\mathrm{AC}}=0$ . With regard to the latter, we start our evaluation by the consideration of the expression for the anion electronegativity (Eqn. (49)) that maximizes the full solvation energy, which is inserted into the reformulated Eqn. (15) in terms of

(52) $$\Delta \Delta E_{\mathrm{AC}}=-\frac{1}{2}\left(\frac{{\left(\delta_{A}^{\max}\right)}^{2}}{\eta_{S}+\eta_{A}}-\frac{\delta_{C}^{2}}{\eta_{S}+\eta_{C}}\right)$$

which then yields

(53) $$\Delta \Delta E_{\mathrm{AC}}=-\frac{\delta_{C}^{2}}{2}\left(\frac{\eta_{S}+\eta_{A}}{{\left(\eta_{C}-\eta_{S}\right)}^{2}}-\frac{1}{\eta_{C}+\eta_{S}}\right)$$

as the corresponding value for the differences in the ion solvation energies under the constraint of maximum full solvation energies. However, the law of matching solvent affinities strictly only holds for $\Delta \Delta E_{\mathrm{AC}}=0$ which is combined with Eqn. (53) and thus yields

(54) $$\eta_{S}^{\mathrm{LMSA}}=\eta_{C}\frac{\eta_{C}-\eta_{A}}{\eta_{A}+3\eta_{C}}.$$

as a necessary condition for the chemical hardness of the solvent in combination with the maximum value for the anion electronegativity. Furthermore, Eqn. (52) in terms of $\Delta \Delta E_{\mathrm{AC}}=0$ also vanishes for $\chi_{C}=\chi_{S}$ , $\eta_{C}\to \infty$ , and $\eta_{S}\to \infty$ . These are global conditions to satisfy the law of matching solvent affinities under the constraint of maximum anion chemical reactivity indices. Hence, the law of matching solvent affinities is valid for infinite solvent or cation hardnesses, as well as for a coincidence between the electronegativities of the cation and the anion in contrast to the assumption of Eqn. (21). If we further assume for Eqn. (54) that $\eta_{C}\gg \eta_{A}$ in agreement with Eqn. (20), one obtains

(55) $$\eta_{S}^{\mathrm{LMSA}}\approx \frac{1}{3}\eta_{C}$$

as a rough estimate for the solvent hardness to satisfy the law of matching solvent affinities. With regard to the large cation electronegativities and moderate solvent chemical hardnesses[^11^, ^12^] and in combination with the relatively good agreement for certain anions with the numerical values obtained through Eqn. (49),^[67]^ it becomes clear why most solvent‐ion pair combinations reveal a reasonable agreement with the law of matching solvent affinities within slight deviations as recently discussed in Ref. [31].

If one combines the maximum electronegativity condition for the solvent (Eqn. (24)) with Eqns. (28) and (29), one obtains

(56) $$\delta_{C}=\chi_{C}-\frac{\chi_{C}+\gamma \chi_{A}}{1+\gamma }=\frac{\gamma }{1+\gamma }\left(\chi_{C}-\chi_{A}\right)$$

and

(57) $$\delta_{A}=\frac{\chi_{C}+\gamma \chi_{A}}{1+\gamma }-\chi_{A}=\frac{\chi_{C}-\chi_{A}}{1+\gamma }.$$

Both expressions are then inserted into Eqn. (15) according to

(58) $$\Delta \Delta E_{\mathrm{AC}}=\frac{{\left(\chi_{C}-\chi_{A}\right)}^{2}}{2\left(\eta_{A}+\eta_{C}\right)}\frac{\eta_{A}-\eta_{C}}{{\left(1+\gamma \right)}^{2}}=0$$

which only vanishes for

(59) $$\chi_{C}=\chi_{A},$$

or

(60) $$\eta_{C}=\eta_{A}$$

as well as for γ→∞ due to $\eta_{C}\to \infty$ . Hence, whenever the solvent electronegativity establishes maximum full solvation energies for given ion pairs, one of the latter conditions needs to be valid for the law of matching solvent affinities. The first two conditions (Eqns. (59) and (60)) have important consequences for the ion pairing behavior and will be discussed in the remainder of this article.

**Figure 3:**
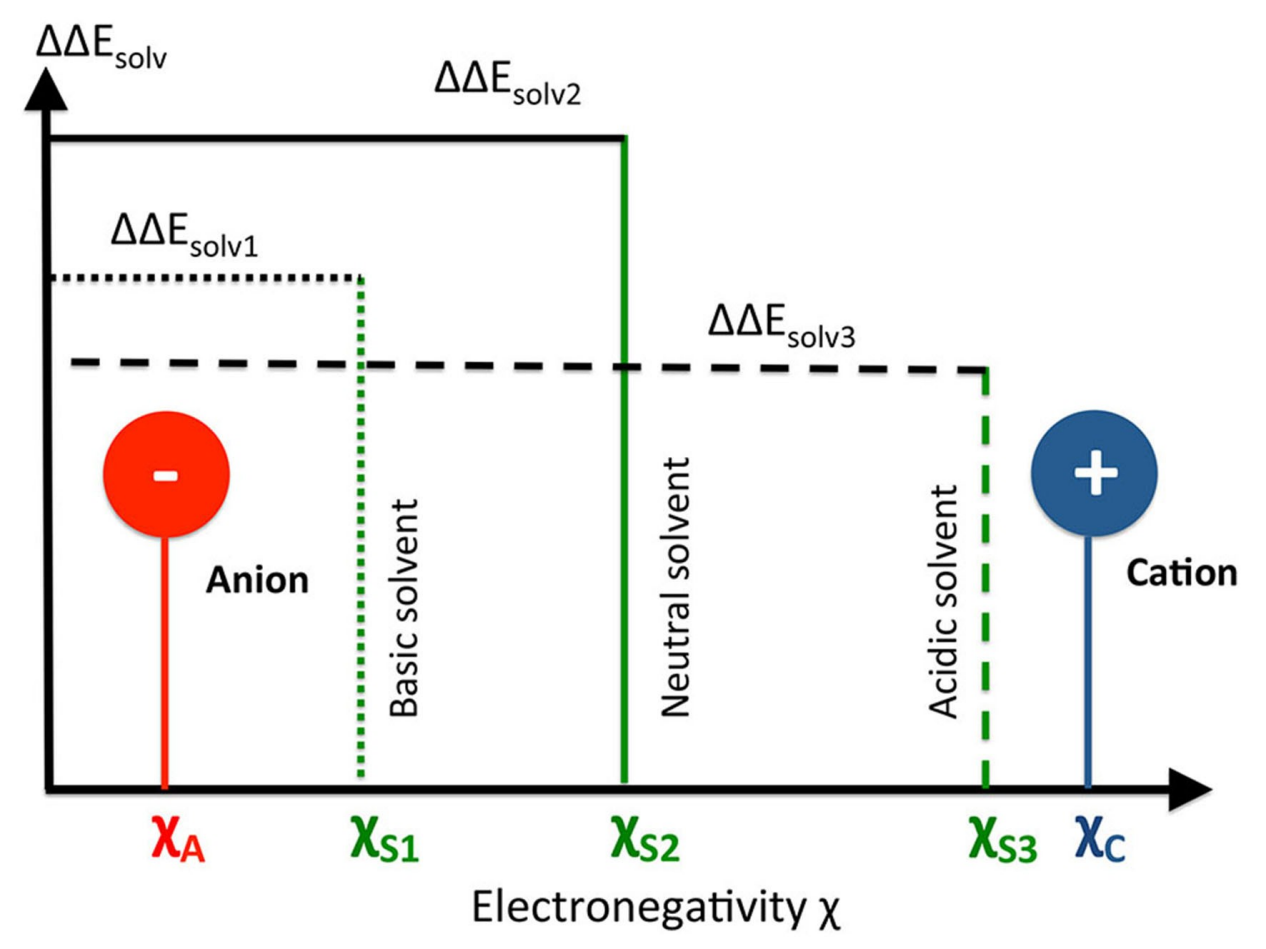
Schematic visualization of the SWAB principle. For ion pairs with electronegativities $\chi_{C}$ and $\chi_{A}$ , the maximum solvation energy $\Delta \Delta E_{\mathrm{solv}2}$ is achieved for the neutral solvent with $\chi_{S2}$ under the condition $\delta_{C}=\delta_{A}$ with $\delta_{C}=\chi_{C}-\chi_{S}$ and $\delta_{A}=\chi_{S}-\chi_{A}$ in terms of $\chi_{S2}=\chi_{S}^{\max}$ according to Eqn. (61). Basic and acidic solvents with $\chi_{S1}$ and $\chi_{S3}$ lead to lower full solvation energies $\Delta \Delta E_{\mathrm{solv}1}$ and $\Delta \Delta E_{\mathrm{solv}3}$ due to $\delta_{C}<\delta_{A}$ and $\delta_{C}>\delta_{A}$ , respectively.

As already discussed, previous assumptions already related the law of matching solvent affinities to empirical concepts like the kosmotropicity as well as the chaotropicity of the ions.[^2^, ^4^, ^7^] Kosmotropes like F^–^ and Li^+^ are usually known as water or solvent structure stabilizers due to their high surface charge density whereas chaotropes like I^–^ and SCN^–^ weaken the local water structure with regard to their large size and their low surface charge density.[^2^, ^3^, ^32^, ^34^] In addition to more refined approaches,[^23^, ^24^, ^27^, ^28^] it was discussed, that ions with comparable water or solvent affinities in terms of comparable solvation enthalpies form the most stable ion pairs. With regard to the previous classification scheme, it is claimed that chaotropic‐chaotropic and kosmotropic‐kosmotropic ion pairs are most stable due to the highest full solvation enthalpies. In contrast, kosmotropic‐chaotropic ion pairs are less stable with often negative free solvation energies. However, with regard to our presented approach and previous findings for co‐solutes,[^68^, ^69^] it comes out that this concept is not fully valid. Instead of identical solvent affinities, our findings reveal that most stable ion pairs form if the electronegativities and chemical hardnesses meet the required conditions. Hence, the chemical reactivity indices like the electronegativities and chemical hardnesses dominate the properties of ions in solution. From a solvent perspective, it comes out that maximum full solvation energies are reached for Eqn. (24) or in the limit of infinite solvent hardnesses. The latter requirement in combination with Eqns. (28) and (29) gives

(61) $$\chi_{S}^{\max}=\chi_{S}+\frac{1}{2}\left(\delta_{C}-\delta_{A}\right)$$

which shows that the chosen solvent electronegativity maximizes the full solvation energy if $\delta_{C}=\delta_{A}$ . Thus, it follows that the assumption of matching solvent affinities has to be replaced by matching acid–base strengths relative to the solvent electronegativity. In consequence, the apparent solvent electronegativity matches the maximum solvent electronegativity in Eqn. (61) according to $\delta_{A}=\delta_{C}$ either for ion pairs with strong acidic cations ($\chi_{C}\gg 0$ ) and strong basic anions ($\chi_{A}\approx 0$ ) as well as for ion pairs with weak acidic cations ($\chi_{C}>0$ ) and weak basic anions ($\chi_{A}>0$ with $\chi_{C}\approx \chi_{A}$ ) in addition to combinations of moderately acidic and basic ions. A comparison with recent results of conceptual DFT calculations[^11^, ^12^] shows that strong kosmotropes like Li^+^ and F^–^ can be identified as strong acidic and basic cations and anions while chaotropes like I^–^ and Cs^+^ reveal weak acidic and basic properties. Hence, the previous assumption of strong ion pair formation for combinations of kosmotropic and chaotropic ions, respectively, can be directly related to a generalized strong and weak acids and bases (SWAB) principle. In this concept, cations and anions are regarded as strong acids and bases whenever $\chi_{C}\gg \chi_{S}$ and $\chi_{A}\ll \chi_{S}$ in contrast to weak acids and bases for $\chi_{C}\approx \chi_{S}$ and $\chi_{A}\approx \chi_{S}$ . Thus, the corresponding electronegativity of the solvent determines the acidity and the basicity of the ions. These conclusions are summarized in Figure 3.

With regard to such considerations, it comes out that a generalized SWAB principle holds for ion pairs in solution with regard to the conditions

(62) $$\chi_{C}\gg \chi_{S}\gg \chi_{A}$$

or

(63) $$\chi_{C}\approx \chi_{S}\approx \chi_{A},$$

respectively. The electronegativity of the solvent therefore acts as a reference value in terms of a three component system to determine the acidity or basicity, respectively, of the cations and the anions. Moreover, the latter ordering scheme is a direct consequence of Eqn. (59). As a result, stable ion pairs are mainly formed by strongly acidic cations with strongly basic anions in relation to the electronegativity of the solvent.

Our equations further provide a rationale for the failure of kosmotropic and chaotropic concepts as recently discussed for neutral co‐solutes.[^68^, ^69^] Previously, it was assumed that also uncharged co‐solutes like urea or trimethylamine‐N‐oxide (TMAO) reveal kosmotropic or chaotropic properties.[^2^, ^32^] In addition to more refined thermodynamic and partitioning arguments,[^16^, ^70^, ^71^, ^72^, ^73^, ^74^] the corresponding consequences were often used to rationalize the structure‐stabilizing and denaturing effects of co‐solutes on proteins.^[2]^ In contrast, recent molecular dynamics (MD) simulations indeed showed that certain structure‐destabilizers like urea reveal a kosmotropic behavior which highlights the fact that chaotropic behavior was never observed for organic co‐solutes.^[68]^ In consequence, it was discussed that most co‐solutes have comparable effects on the water structure and dynamics^[68]^ such that kosmotropic and chaotropic concepts for co‐solutes fail to rationalize the effect on protein structures. Notably, the corresponding co‐solutes are of roughly similar size and the gap between HOMO and LUMO energy values is rather small, such that their electronegativities do not reveal significant differences in the acidity or basicity. With regard to the definition of the reaction energy between the co‐solute and the solvent (Eqn. (10), one thus would assume comparable interaction strengths, such that a previously observed identical behavior for all co‐solutes becomes reasonable.[^68^, ^69^]

Moreover, single‐atom species with similar nuclear charges and identical electronic configurations tend to have similar hardnesses, which can be rationalized by their similar size and value of the HOMO and LUMO energies. Hence, Eqn. (60) is a manifestation of the hard–soft acid and base (HSAB) principle,[^47^, ^49^, ^56^] since it implies that ions with comparable hardnesses form the strongest ion pairs.

Furthermore, the previous outcomes also have important consequences for the interpretation of ion‐solvent complexes including charge transfer. With regard to previous discussions,^[39]^ the amount of transferred charge between two reacting species A and B can be defined as

(64) $$\Delta N_{\mathrm{AB}}=-\frac{\chi_{B}-\chi_{A}}{\eta_{A}+\eta_{B}},$$

with the condition $\Delta N_{\mathrm{AB}}=-\Delta N_{\mathrm{BA}}$ to ensure electroneutrality. Hence, it follows for the charge transfer between the solvent and the cation or the anion, respectively, that

(65) $$\Delta N_{\mathrm{CS}}=-\frac{\delta_{C}}{\eta_{C}+\eta_{S}}$$

and

(66) $$\Delta N_{\mathrm{AS}}=\frac{\delta_{A}}{\eta_{A}+\eta_{S}}.$$

Noteworthy, a sufficient condition for the LMSA is that $\delta_{C}=\delta_{A}$ (Eqn. (59)) which, together with Eqn. (51) implies that when the charge transfer from/to the solvent to/from the anion/cation are comparable, in other words, when $\Delta N_{\mathrm{CS}}\approx \left|\Delta N_{\mathrm{AS}}\right|$ , the LMSA will hold. With regard to these findings, one could interpret solvated ions as solvent‐ion complexes with charge transfer, which was already observed for simple ions in water as well as more complex deep eutectic electrolytes.[^64^, ^65^, ^75^]

### 3.5 Shape of Volcano Plots

**Figure 4:**
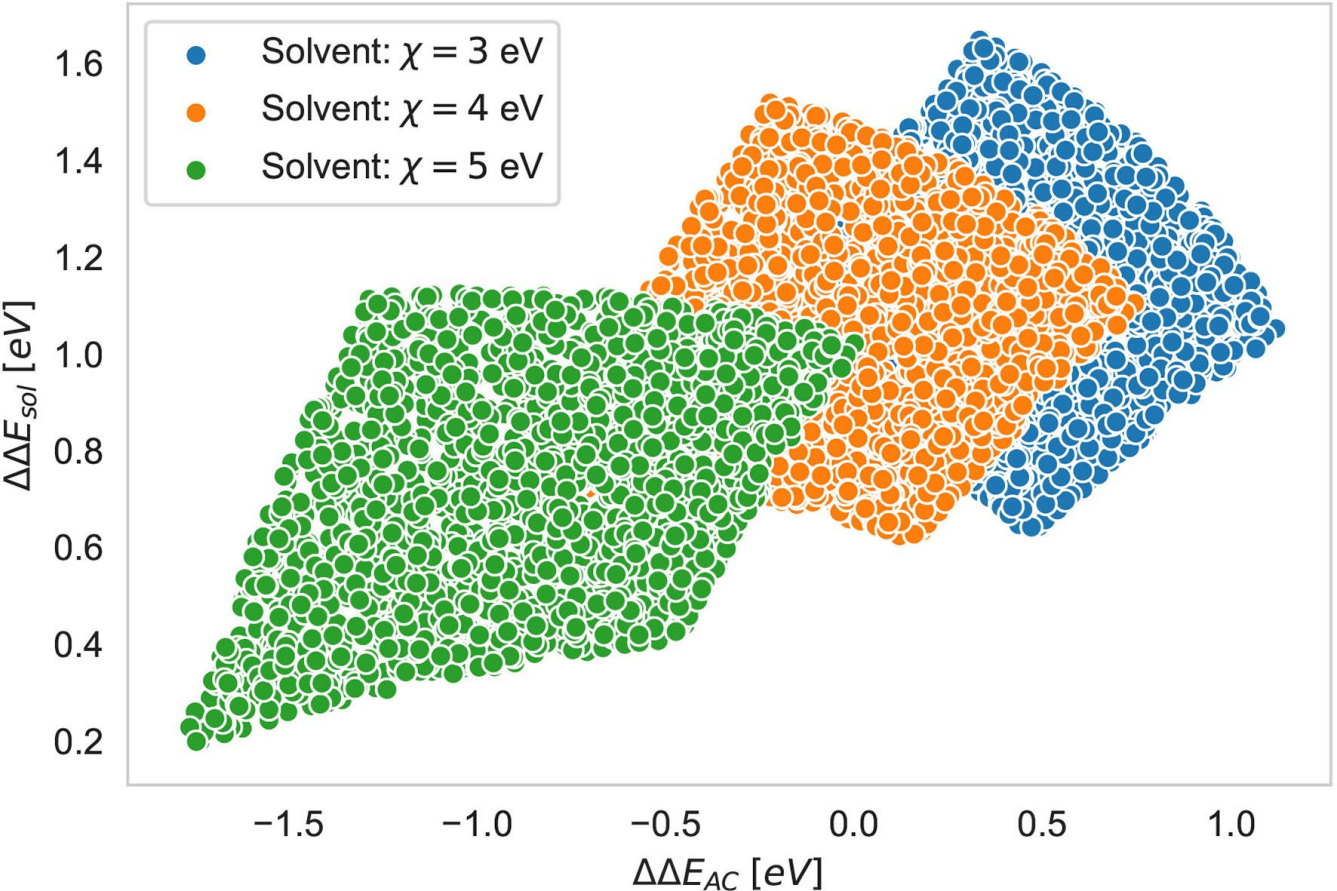
Full solvation energy $\Delta \Delta E_{\mathrm{solv}}$ and difference in anion and cation solvation energies $\Delta \Delta E_{\mathrm{AC}}$ for 2000 ion pairs in a solvent with chemical hardness $\eta_{S}=5$ eV and varying electronegativities between $\chi_{S}=3$ eV–5 eV as denoted in the legend.

Under the assumption of fixed ions with given electronegativities and hardnesses, one may ask how the shape of the volcano plot changes for various solvents and the corresponding chemical hardnesses and electronegativities. This question is closely related to experimental conditions, where a set of well‐defined ion pairs is usually brought into contact with various solvents.^[31]^ In order to study the influence of varying solvent chemical reactivity indices, 2000 artificial cation and anion pairs (electronegativity values between $\chi_{C}=8$ eV –10 eV and $\chi_{A}=0$ eV–2 eV, hardness values between $\eta_{C}=10$ eV –12 eV and $\eta_{A}=1$ eV –3 eV) were sampled from a random uniform distribution. Herewith, we strictly follow the same methodology which was recently used to reproduce volcano plots in good agreement with experimental results.^[12]^ As a first step, the chemical hardness of the solvent was kept constant with $\eta_{S}=5$ eV and the electronegativity was varied from $\chi_{S}=3$ eV –5 eV. As can be seen in Figure 4, the upper borders of the resulting plot reveal the typical volcano‐shape behavior which was already observed for the experimental results in Figure 1 and in previous publications.[^12^, ^31^] Notably, the resulting squares highlight the large space of potentially accessible values for certain ion pair combinations which rationalizes the fact that most ion pairs in distinct solvents do not show a linear behavior with constant slope.[^12^, ^31^] Moreover, with increasing electronegativity of the solvent, one can observe a change of the steepness for the upper borders of the squares. Hence, for solvents with lower electronegativities, the upper right border shows a stronger decrease when compared to the upper left border while this behavior changes for higher solvent electronegativities. Furthermore, it can be clearly seen that the center of the square moves from positive to negative $\Delta \Delta E_{\mathrm{AC}}$ values with increasing solvent electronegativities. With regard to our previous discussion of Eqns. (12), (13) and (14), it becomes clear that lower solvent electronegativities $\chi_{S}<\chi_{s}^{\max}$ favor the solvation of the cations while larger values $\chi_{S}>\chi_{s}^{\max}$ reveal a stronger attraction to the anions. Due to mean values for the chemical reactivity indices in terms of uniform distributions, one obtains for Eqn. (24) a value of $\chi_{S}^{\max}=3.43$ eV which thus underpins our previous interpretations. Thus, the right square with $\left\langle \chi_{S}\right\rangle =3$ eV is clearly dominated by cation‐solvation effects as also expressed by $\left\langle \Delta \Delta E_{\mathrm{AC}}\right\rangle >0$ with $\left\langle \Delta E_{\mathrm{CS}}\right\rangle \ll \left\langle \Delta E_{\mathrm{AS}}\right\rangle$ which changes for higher electronegativities to anion‐dominated solvation in terms of $\left\langle \Delta \Delta E_{\mathrm{AC}}\right\rangle <0$ due to $\left\langle \Delta E_{\mathrm{CS}}\right\rangle \ll \left\langle \Delta E_{\mathrm{AS}}\right\rangle$ . With regard to the experimental values in Figure 1, one can assume that most solvents are located below their maximum electronegativity. Comparable conclusions can be also drawn with regard to recent results for the electronegativities and chemical hardnesses of the interacting ions and solvents as shown in Refs. [11, 12, 67].

Notably, previous works already demonstrated that the presented approach provides a qualitative agreement between computed and experimental data for various ion pairs in distinct protic and aprotic solvents.^[12]^ Hence, the conclusions on the shape of volcano plots as well as the general properties of ion pair‐solvent combinations are also supported by previous calculations and can be directly applied to experimental findings.^[31]^ With regard to the shape of the volcano plot for various ion pairs and distinct solvent shown in Figure 1, it can be concluded that the electronegativity of most solvents is significantly lower when compared to the electronegativity of the cation. Thus, it follows that $\delta_{A}\ll \delta_{C}$ which implies that the solvation energy is mainly dominated by the energetic contributions $\Delta E_{\mathrm{CS}}\ll \Delta E_{\mathrm{AS}}$ through the solvation of the cation. Corresponding conclusions can also be drawn with regard to the results for distinct solvents shown in Ref. [31]. Notably, these findings are in good agreement with previous calculations, ^[11]^ where it was shown that for most protic and aprotic solvents the relation $\delta_{A}\ll \delta_{C}$ remains valid.

## 4 Summary and Conclusion

By means of conceptual DFT calculations, we provide a rationale for specific ion effects in distinct solvents. The corresponding expressions are used to study the underlying principles of volcano plots as well as the law of matching solvent affinities. In more detail, we show that the mechanisms leading to most and least stable ion pairs depend crucially on the electronic properties of the ions as well as the solvent in terms of electronegativities and the chemical hardnesses. Further outcomes of our study imply that the law of matching solvent affinities is only applicable when certain conditions are satisfied. Moreover, we have shown that the stability of ion pairs can be related to a generalized strong–weak acid–base (SWAB) principle. Here, the solvent acts as a reference medium which determines the acidity and the basicity of the ions. Notably, our expressions allow us to reproduce the specific shape of volcano plots and thus to broaden the theoretical understanding of such effects.

Our analytic expressions can be used to analyze the properties of ions in solution, their pairing tendencies and shed new light on specific ion effects. With regard to the fact that all electrostatic interactions are ignored, one can assume that long‐range as well as higher order dispersion effects seem to be of minor importance. Such a crucial approximation can be related to a short‐ranged dielectric screening mechanism by polar solvents such that all relevant electrostatic length scales are smaller than a few nanometers.^[17]^ In contrast, one may expect that the corresponding contributions change in presence of apolar solvents with low dielectric constants like chloroform. However, previous articles already showed that electrostatic and higher order interaction mechanisms also can be taken into consideration straightforwardly.[^49^, ^50^] Thus, most of the ion solvation effects can be attributed to electronic perturbation which is the reason for the occurrence of solvation bonds as well as solvent‐ion charge transfer complexes.[^11^, ^17^, ^65^] Moreover, one can also assume to apply a comparable approach for the study of closely related problems like the ion‐specific adsorption behavior at solid‐liquid interfaces or around polyelectrolytes. In presence of homogeneous surfaces or homopolyelectrolytes formed by one single molecular species, it can be expected that the corresponding approach may provide useful results to rationalize the observed accumulation behavior.[^10^, ^18^] In summary, our framework adds an important and straightforward alternative view on solvation effects as well as the occurrence of specific ion effects in distinct solvents.

## Conflict of interest

The authors declare no conflict of interest.
